# A Rough Set-Based Model of HIV-1 Reverse Transcriptase Resistome

**DOI:** 10.4137/bbi.s3382

**Published:** 2009-10-05

**Authors:** Marcin Kierczak, Krzysztof Ginalski, Michał Dramiński, Jacek Koronacki, Witold Rudnicki, Jan Komorowski

**Affiliations:** 1 The Linnaeus Centre for Bioinformatics, Uppsala University BMC, Box 598, Husargatan 3, SE-751 24 Uppsala, Sweden; 2 Interdisciplinary Centre for Mathematical and Computational Modelling, Warsaw University, ul.Żwirki i Wigury 93, 02-089 Warszawa, Poland; 3 Institute of Computer Science, Polish Academy of Sciences, ul. J.K. Ordona 21, 01-237 Warszawa, Poland. Email: jan.komorowski@lcb.uu.se

**Keywords:** viral proteomics, bioinformatics, HIV-1 drug-resistance, viral complexity, resistance model

## Abstract

Reverse transcriptase (RT) is a viral enzyme crucial for HIV-1 replication. Currently, 12 drugs are targeted against the RT. The low fidelity of the RT-mediated transcription leads to the quick accumulation of drug-resistance mutations. The sequence-resistance relationship remains only partially understood. Using publicly available data collected from over 15 years of HIV proteome research, we have created a general and predictive rule-based model of HIV-1 resistance to eight RT inhibitors. Our rough set-based model considers changes in the physicochemical properties of a mutated sequence as compared to the wild-type strain. Thanks to the application of the Monte Carlo feature selection method, the model takes into account only the properties that significantly contribute to the resistance phenomenon. The obtained results show that drug-resistance is determined in more complex way than believed. We confirmed the importance of many resistance-associated sites, found some sites to be less relevant than formerly postulated and—more importantly—identified several previously neglected sites as potentially relevant. By mapping some of the newly discovered sites on the 3D structure of the RT, we were able to suggest possible molecular-mechanisms of drug-resistance. Importantly, our model has the ability to generalize predictions to the previously unseen cases. The study is an example of how computational biology methods can increase our understanding of the HIV-1 resistome.

## Introduction

More than two decades have passed since the discovery of HIV, the causative agent of AIDS. Numerous groups focused their research on understanding the details of HIV life cycle and on developing efficient antiviral therapies. Unfortunately, the high rate of replication combined with the high mutability of the virus leads to the rapid emergence of drug-resistant strains efficiently undermining the efforts to stop the AIDS pandemic. Currently, there are some 7,000 new HIV infections reported worldwide every day. In total, more than 30 million people in both the developed and the developing countries are HIV-positive.^1^ About 10^9^ virions are produced in an infected individual every day and it has been estimated that each possible single-point mutation arises 10^4^–10^5^ times in this population.^2^ While some mutations result in the production of functionally-impaired viruses, other lead to the emergence of drug-resistant forms.

Reverse transcriptase (RT) is one of the viral enzymes that are required for successful replication. The RT catalyzes reverse transcription, a process of transforming single-stranded viral RNA into double-stranded viral DNA. The viral DNA is later incorporated into the host genome and it re-programs the host cell to produce new viral particles that undergo maturation, bud off and infect new cells thus completing the viral life-cycle. In peripheral blood lymphocytes the maturation occurs after viral release while in macrophages it takes place prior to the release, within the cell, in the multivesicular bodies. Not unlike the other enzymes in the family of reverse transcriptases, the HIV-1 RT lacks proof-reading activity which, combined with the high replication rate of the virus and the RT-mediated recombination, leads to the rapid emergence of HIV mutants. Many of these mutants are drug-resistant. The first antiviral therapies were targeted against the RT and this enzyme still remains one of the most common targets for anti-HIV drugs. An initial hope that followed the introduction of AZT (Zidovudine), the first anti-viral agent targeting HIV, has been quickly shattered by the rapid emergence of drug-resistant viruses. Among the 25 drugs currently used in HIV therapy, 12 attempt at inhibiting the RT enzyme.

There exist two groups of RT inhibitors, namely the nucleoside/nucleotide RT inhibitors (NRTI) and the non-nucleoside RT inhibitors (NNRTI). The former ones mimic dNTPs, the ordinary RT substrates but due to the lack of the 3’-OH group in the ribose ring they inhibit DNA chain elongation immediately after being incorporated. The mode of action of the NNRTI drugs is somewhat different since they bind in the so-called NNRTI-binding pocket of the RT and induce conformational changes that terminate the synthesis of the viral DNA.

Various attempts have been undertaken to associate particular mutations in the RT sequence with the drug resistance level. Often, however, it is not a single mutation, but rather a non-linear combination of different mutations that leads to drug resistance. This increases the complexity of the problem and various machine learning techniques have been used in order to predict resistance from RT sequence. Drăghici and Potter^3^ have used neural networks to build a predictive model of HIV drug resistance to RT inhibitors. The commonly used Geno2Pheno tool^4^ relates sequence to resistance by using regression models. An international panel of experts semiannually releases a set of rules for predicting resistance.^5^ Similar approach has been used by Johnson et al^6^ Garriga and Menéndez-Arias^7^ released a tool that uses the available sets of expert-derived rules to predict resistance. In their interesting studies, Rhee et al^8^ use five different statistical learning methods (decision trees, neural networks, support vector regression, least-squares regression and least-angle regression) to model sequence-resistance relationship in HIV-1. A fresh and stimulating approach to the problem is presented in Kjaer et al^9^ where the authors propose to represent protein sequences in terms of physicochemical properties of amino acids. Recently, Prosperi et al^10^ published an interesting comparison of linear and non-linear machine learning techniques used in HIV resistome research. They conclude that fully data-driven models derived from large-scale data are promising as antiretroviral treatment decision support tools and postulate complementing sequence data sets with patient-derived data such as treatment history.

Although the existing models were able to predict HIV-1 resistance to RT inhibitors, none of them provided any deeper insight into the underlying mechanisms in a physicochemical sense. There was also a lack of a method that would be able to predict resistance caused by a previously unseen mutation. In this paper we attempted at filling this gap by developing a computational model of HIV-1 resistance to several RT inhibitors. Rather than looking at mutating amino acids, we based our model on local physicochemical properties of a protein sequence. This approach, combined with the Monte Carlo feature selection and the rough set theory resulted in an interpretable high quality model of the RT resistome. The model consists of a number of general IF-THEN rules associating changes in the physicochemical properties of RT-sequence with drug resistance level, e.g.:

IF (polarity at site 101 = (−∞, 2.100)) AND (normalized freq. of turn at site 190 = [0.045, ∞])

THEN resistant to Nevirapine

This makes the model easy-to-interpret and generative and lets us believe that the presented approach will contribute to the development of new, more potent antiretroviral drugs.

## Materials and Methods

### Data

We used publicly available data obtained from Stanford HIV Drug Resistance Database.^8^ For each of the examined drugs we extracted a number of amino acid sequences of the HIV-1 RT p66 subunit. Each sequence in the database has been annotated with the resistance value relative to the HXB2 wild-type strain. Since Zhang et al^11^ have demonstrated that the Monograms PhenoSense is more reliable than other drug-resistance-testing assays and that it produces highly reproducible results, we used only the sequences with the resistance value determined using this method. In total, there were 781 sequences of the p66 subunit (91% of them complete within the first 240 aa sites, 31% of them complete within all the 560 aa sites) that we could use for constructing data sets. Following the established clinical practice, we labeled each sequence as “susceptible”, “moderately resistant” or “resistant”. We used cut-off values for the discretization as described in Rhee et al.^8^ The detailed distributions of the resistance classes per drug are presented in Table 1.

### Description of sequences

Kjaer et al^9^ have used 544 different physicochemical properties of amino acids obtained from the aaIndex database^12^ to describe HIV-1 protein sequences. Although we used the descriptors from the same database, our approach is different. Rather than constructing a large number of data sets, each based on a single physicochemical property, we constructed one data set per each antiviral drug and described each amino acid in a sequence by a vector of biologically relevant and interpretable properties. Following procedure described by Rudnicki and Komorowski,^13^ we extracted a number of biologically-meaningful descriptors from the aaIndex database.

First, we selected descriptors that are representative for three broad biophysical categories:

1. Transfer free energy from octanol to water^14^ for hydrophobicity;
2. Normalized van der Waals volume^15^ for size;
3. Isoelectric point^16^ for charge.

**Figure 1.**
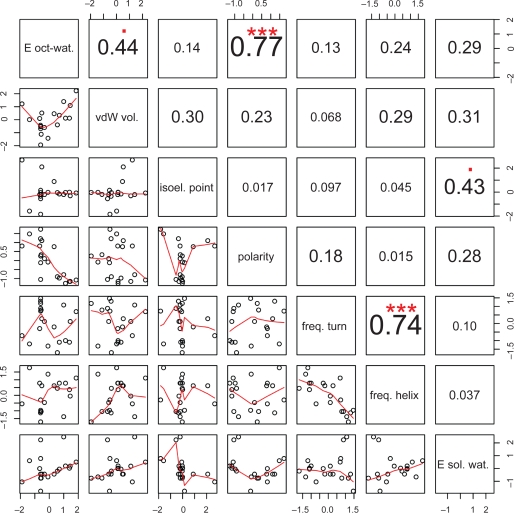
Correlation matrix of physicochemical descriptors. The lower triangle contains bivariate scatter plots with a fitted line. The actual absolute values of the correlation are provided. The significance levels of the correlation are encoded in the following way: p <= 0.001(***); p <= 0.01(**); p <= 0.05(*); p <= 0.1(.).

These properties were fixed during the simulated annealing run. Than we added randomly four different properties and computed the sum of the r-square for all pairs of this set, which was used as a pseudo-energy measure. A single move in the simulation consisted of replacing one of the four random properties. Moves leading to the decrease of pseudo-energy were always accepted, and moves leading to the increase of pseudo-energy were accepted with the probability:

p=exp(−DE/(kT))

where DE is the the increase of pseudo-energy, T is a pseudo-temperature and k is a scaling constant. The pseudo-temperature was slowly decreasing during simulation, from 1000 to 1, and the scaling constant was selected by trial and error. Ultimately, we selected seven relatively low-correlated (cf. Fig. 1) physicochemical descriptors that are presented in Table 2.

The selected properties let us represent each naturally occurring amino acid as a unique point in the coordinates frame spanned by them. After the description, each amino acid sequence in the data set was represented by 3,920 properties (560 aa × 7 properties). We described each site in an aa sequence as a difference between the vector representing the wild-type and the vector representing the observed amino acid. Therefore, if no mutation was observed at all, the site was described by the vector of seven zeroes. The final data sets were the ensembles of the described sequences annotated with the drug resistance values.

### Monte Carlo feature selection

In order to select only the attributes (here the properties of 560 amino acids) that significantly contributed to drug resistance, we applied Monte Carlo Feature Selection (MCFS) method as described in Dramiński et al.^21^ In short, MCFS relies on the construction of a large number of decision trees. Trees are trained on different random subsets of attributes and different random subsets of objects. More precisely, out of all *d* features, we select *s* random subsets of *m* features, *s* and *m* being fixed, *s* being large and *m* ≪ *d,* and for each subset of features, *t* trees are constructed and their performance is assessed. Each of the *t* trees in the inner loop is trained and evaluated on a different, randomly selected training and test data sets. The evaluation results obtained from all *s.t* trees let one build a ranking of features reflecting their importance or, in other words, their discriminative power. In due course, the most informative features are selected with the help of a Student’s t-test.

In this way, all non-informative features were removed from the initial data set. The results of the feature selection are presented in tables: Table 3–Table 10.

For the sake of comparison, the process of attributes-ranking differs between Breiman’s random forests (RF)^22^ and MCFS. In RF, the ranking is obtained by reshuffling the values of an attribute and observing the change in the quality of classification. In MCFS randomization test is done in a standard way by reshuffling decision labels. The importance of an attribute is determined by looking at the weighted accuracy related to randomization test-derived background. Another important difference between MCFS and RF is that while in the former individual trees are built on training samples drawn without replacement from the original set of samples (and are evaluated on the remaining samples) in the latter bootstrap techniques are used which rely on sampling with replacement.

We perform feature selection on the whole entire data sets prior to splitting them into the training set and the test set. In our previous work,^21^ we argue in detail and show by examples that the MCFS provides a possibly objective ranking of features, independent of a classifier to be later used and pertaining only to the classification problem *per se.* In particular, using the MCFS does not lead to overfitting when proper classification is performed. At the same time, to benefit the most from the application of the MCFS, it should be performed on the largest available set of examples.

### Rough sets

Rough set theory described in Pawlak^23^ has been introduced in the early eighties. It constitutes a mathematical framework particularly suitable for dealing with imprecise and incomplete data. In the rough set-based machine learning a set of minimal decision IF-THEN rules is inferred from a number of labelled examples. These rules constitute a model that can be used for assigning class labels to the previously unseen objects. The IF part of a rule is a conjunction of feature values and the THEN part is a disjunction of class labels. We used the ROSETTA^24^ implementation of the rough set theory in order to learn a number of IF-THEN rules that associate the MCFS-selected physicochemical properties of the amino acids of the HIV-1 RT with the resistance level.

As it is required by the rough sets approach that all the features take discrete values, we first applied the entropy scaler and the equal frequency binning discretization algorithm. The process of inferring minimal sets of features (reducts) is computationally expensive. We used a genetic algorithm, a heuristic approach to finding approximate reducts. The obtained reducts let us infer a number of IF-THEN rules that link minimal combinations of amino acid properties with a resistance level. In order to make the model even more general, we applied a rule-generalization algorithm as described by Mąkosa.^25^ In short, a general rule is obtained by merging similar or partially redundant rules and on relaxing constraints imposed by them. For instance the following three rules (abbreviations explained in Table 2):

IF P101 polarity ((–∞, 2.100)) AND P190 freq. turn ([0.045,∞)) AND P179 freq. turn((−∞, 0.70)) THEN resistant to Nevirapine

IF P101 polarity([−1.800, 2.100)) AND P190 freq. turn([1.40,∞)) THEN resistant to Nevirapine

IF P101 polarity((−∞, −0.500)) AND P190 freq. turn([0.045, 1.50)) AND P179 freq. turn((−∞, 0.70)) THEN resistant to Nevirapine

are partially redundant and can be merged into one rule:

IF P101 polarity ((−∞, 2.100)) AND P190 freq. turn ([0.045,∞)) AND P179 freq. turn((−∞, 0.70)) THEN resistant to Nevirapine

Since the removal of the P179 freq. turn ((–∞, 0. 70)) part has very little effect on the accuracy of the rule, further simplification can be applied which results in the final rule:

IF P101 polarity ((−∞, 2.100)) AND P190 freq. turn ((0.045,∞)) THEN resistant to Nevirapine

By using general rules we minimized the risk of overfitting our model to the training data. The ensemble of this rules constitutes a model that can be used to predict resistance of new HIV-1 strains. Typically all the rules that constitute the model vote for the final decision. A threshold defining a minimal amount of votes necessary to label an object with a decision may result in multiple decisions for the same object. We would like to emphasize that the rules used by the model are inherently descriptive and can easily be analyzed by a domain expert. The description of the data is presented in Table 1. Table 12 provides the detailed description of the models.

### Validation

The validity of each model was determined in 10-fold cross-validation and in the so-called randomization test. In addition, the predictive quality of each general model was verified using an external test set. First, we randomly divided each data set into a training set and an external test set. Each training set contained 80% of the sequences from the original data set and the remaining 20% of the sequences constituted the external test set. Both the training and the test set had the same distribution of the decision class (resistance) as the original data. Subsequently, we performed 10-fold cross-validation on the training set. The training data were randomly divided into ten subsets of equal size, D_i_, i = 1, 2, …, 10. We then generated ten new training sets of sequences (N_i_) by sequentially removing one of the D_i_ subsets from the original training set. Thus, the N_1_ data set contained all the data but the D_1_ subset, the N_2_ data set contained all the data but the D_2_ and so forth. Thereafter we used each of the N_i_ training sets to build a rough set-based classifier. The classifier was then used to classify the objects from the remaining D_i_ subset. Therefore each sequence from the original data set was present once in a test set and nine times in a training set. In order to assess the probability that the obtained results could have been generated by random data, we constructed additional 1000 data sets per model by randomly permuting the decision in the original data set. Thus, we broke correspondence between the sequence and the resistance value. Each of the 1000 randomized data sets was evaluated using 10-fold cross-validation. Ultimately, we were using all the sequences from the original data set to train a rough set-based classifier and validated the predictions on the external test set.

The performance of the models was validated using prediction accuracy and the area under the ROC (or Receiver Operating Characteristic) curve AUC. The accuracy, equal to a fraction of correctly classified sequences, was measured by its mean value for the cross-validated experiments and, finally, by its measurement on the external test. The AUC was measured by its mean for the cross-validated experiments.

For a two-class classification task, the ROC curve accounts for an uneven distribution of the decision classes in the original data set and visualizes the behavior of the classifier at different sensitivity to specificity ratios. *Sensitivity* is defined as a ratio between true positive predictions and the total number of positives. *Specificity* is a ratio between true negative predictions and the total number of negative examples. The ROC curve is constructed by plotting sensitivity vs. 1-specificity. The AUC value is an integral over the ROC curve. For a perfect binary classifier we have AUC = 1.0 whereas for a random classifier AUC = 0.5. Since in our case the decision takes three distinct resistance values: “susceptible”, “moderately resistant” and “resistant”, we provide a separate AUC value for each class by treating the two remaining classes as one. For instance, to calculate an AUC value for the class “susceptible”, we consider both the “moderately resistant” and the “resistant” as a new “non-susceptible” class.

At last, we used the results of the randomization tests to compute a kind of p-values, i.e. the probability that the relationships found in the original data arose by pure chance. Our computations were based on the assumption that the AUCs obtained in the randomization test are normally distributed. The normality was assessed by examining the so-called Q-Q plots and applying Shapiro-Wilk test for normality. Subsequently we used Student’s t-test to obtain the p-values.

In addition, we compared the performance of our models with the performance of their standard decision tree-based counterparts with mutations represented by one-letter aa codes. We used J48 algorithm as provided in the WEKA^26^ suite to derive the decision tree models.

## Results and Discussion

Application of the Monte Carlo feature selection method combined with a rough set-based approach resulted in statistically sound, interpretable and generative rule-based models of the RT sequence-resistance relationship. The models can be used to predict HIV-1 resistance to six different NRTI drugs and two NNRTIs. By representing mutating amino acids in terms of physicochemical changes, the models gained generality and can be used to predict resistance for previously unseen mutants. Let us assume that only the following amino acids have been observed at site 101: A, E, H, K, P, Q, R, S, insertion, and that this observation led to the following rule:

IF (polarity at site 101 = (−∞, 2.100)) THEN resistant to Nevirapine

Now, if the model is asked to predict whether a newly observed mutation to asparagine at site 101 will result in drug resistance, the polarity value for asparagine, (polarity_N_ = 11.60) will be substituted to the rule and the prediction will be “Resistant to NVP”.

At the first step, each RT sequence was represented by 3,920 properties. Application of the MCFS led to a significant reduction of this number (see Table 3–Table 10). It was already at this point that we have discovered that mutations at several, previously unnoticed sites contribute to drug resistance. There are 5 such sites for Abacavir, 5 for Didanosine, 4 for Lamivudine, 8 for Stavudine, 6 for Tenofovir, 6 for Zidovudine, 10 for Delavirdine and 5 for Nevirapine. Apart from these, there are several sites where mutations were previously associated with resistance to some drugs, but our results suggest that also resistance to other drugs may be induced by them. We speculate that mutations at the newly discovered sites may be either directly responsible for drug-resistance or may play compensatory role by accompanying other drug-resistance mutations and diminishing their negative effects, e.g. the decreased replication rate. Table 11 presents sites that are included in various sets of rules for predicting drug resistance^5^,^6^ but were not selected as significant by the MCFS method. The missed sites are either underrepresented in the data sets or their influence on drug-resistance is much weaker than previously assumed. This issue has to be investigated further.

Following the feature-selection step, we applied rough set approach to build rule-based models of HIV-1 resistance to drugs. We used two different sets of parameters leading either to very specific or to more general rules that underly a model. Prior to model-building, we excluded 20% of the available examples from each data set in order to use them for independent validation. We used the remaining data for model-construction. We validated our models in 10-fold cross-validation and used area under ROC curve to measure their performance. All the models showed good results with accuracy varying from 69% for Delavirdine to 89% for Lamivudine when using specific sets of rules and from 69% for Delavirdine to 88% for Lamivudine when using generalized rules. Similarly, the corresponding AUC values were high in the majority of the models (cf. Table 12). In some cases, e.g. the resistance-to-Nevirapine model that was based on general rules, we observed low AUC values for the “moderately resistant” class. This may be due to the fact that the artificially set threshold values and the arbitrary split into three resistance classes is not completely reflected in real mutation patterns. Generalization of the rules did not lead to any significant deterioration of the classification quality.^25^ At the same time it reduced the number of rules by an order of magnitude. Models built on general rules are smaller, less sensitive to overtraining and easier to analyze.

Finally, we validated each model on an external test set (20% of the available examples). In addition, we compared the performance of our models to the standard decision tree-based models. The decision trees performed similarly to their rough set-based counterparts but at the same time they were less stable. The decision tree-based models derived with no feature selection step loose generality and an important interpretational layer. The results are summarized in Table 12.

We also compared our model based on generalized rules with the model described by the domain expert rules^5^ (cf. Table 13). For both sets of rules, we computed coverage and accuracy. In the case of the domain expert rules, we could use the entire data sets for the computation while in the case of the rough set model, we used only the test sets to avoid the possible bias caused by the fact that the rules were derived from the training data. Therefore for our model, we provide only a pessimistic estimates of accuracy and coverage. While accurate, expert rules are applicable only to a very limited fraction of examples. The generalized rules that underlie our model have significantly higher coverage.

Importantly, our generalized rules are conjuncts of the values (intervals of values) of physicochemical properties of amino acids. This allows seeing which amino acids fulfill the criteria imposed by a given rule, also when such amino acids were not represented in the training set. Given the following rule:

IF P101 polarity ((−∞, 2.100)) AND P190 freq. turn ([0.045,∞)) THEN resistant to Nevirapine

we can easily find which amino acids satisfy the conditions and substitute them into the rule:

IF P101(any of: D, E, H, K, N, Q, R) AND P190(any but: A, G, N, P, Y) THEN resistant to Nevirapine

Even though asparagine (N) was not observed at site 101 in the available data, our general model is able to foresee that an occurrence of such a mutation may result in the acquisition of resistance. Such an approach already proved to be successful in revealing mechanisms underlying resistance to protease inhibitors.^27^

**Figure 2.**
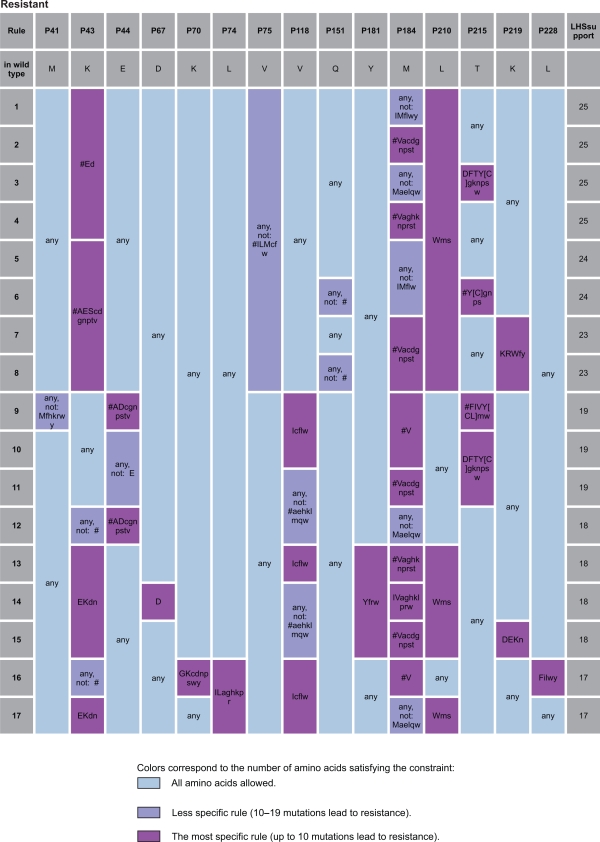
The strongest rules determining resistance to Abacavir. Amino acids are encoded using standard one-letter abbreviations. # indicates insertion of any type; “AA” is an amino acid observed in the data in the given resistance class; “[AA]” represents an amino acid observed in the data, but in the other resistance class and “aa” denotes an amino acid not observed in the data. “LHS support” is a number of examples satisfying the rule.

**Figure 3.**
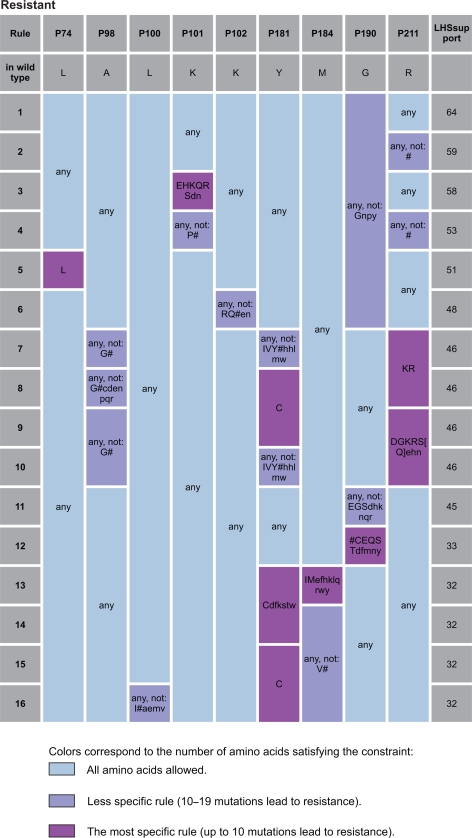
The strongest rules determining resistance to Nevirapine. Amino acids are encoded using standard one-letter abbreviations. # indicates insertion of any type; “AA” is an amino acid observed in the data in the given resistance class; “[AA]” represents an amino acid observed in the data, but in the other resistance class and “aa” denotes an amino acid not observed in the data. “LHS support” is a number of examples satisfying the rule.

Figure 2 and Figure 3 present an instance of analysis of the strongest rules determining resistance to Abacavir and Nevirapine respectively. For more details see Supplementary Material, Figure S1–S7.

All the remaining sets of rules were included in the online supplementary material.

Detailed analysis indicates that although amino acids at these newly discovered positions interact directly neither with nucleic acid nor with the ABC triphosphate (ABCTP), the detected mutations may disturb the complex network of hydrophobic and polar interactions responsible for the stability of the tertiary structure. This may lead to subtle structural changes in the relative orientation of the domains and active site architecture, preventing ABCTP binding in a catalytically competent configuration. However, it seems that these small structural changes do not prevent the ability of a drug-resistant enzyme to incorporate normal nucleotides in the catalyzed reaction.

There are 10 sites (98, 100, 101, 103, 106, 108, 181, 188, 190 and 230) that experts have associated with the resistance to Nevirapine. The model finds all these important (except the 108 and the 230 site) and pinpoints six other sites as significant (102, 211, 357, 379, 401 and 468). None of these was previously associated with resistance. Additionally, sites 74 and 184 associated so far only with resistance to NRTI drugs and site 179 previously connected to resistance to the other NNRTI drugs, transpired to play significant role in acquiring the resistance to Nevirapine.

Since the training data does not contain any information on the history of treatment, some of the newly discovered sites might have emerged as a result of the past therapies. For instance, sites 74 and 184 known to contribute to resistance to NRTI drugs were selected as important to the resistance to Nevirapine which is a NNRTI drug. Therefore their role in the resistance to Nevirapine should be further investigated.

Similarly, sites that are often mutated in other HIV subtypes^28^–^30^ (e.g. 35, 43, 122, 123, 135, 200, 211) should be treated with caution. While Kearney et al^28^ consider sites 35, 83, 122, 123, 135, 200 and 211 as “non-resistance polymorphic”, Kantor and Katzenstein^29^ suggest that mutations at these sites (in particular 43 and 211) may play a significant role in drug resistance evolution and increase viral fitness. Site 118 that our method selected as important to resistance to some NRTI drugs was previously considered important but in 2005 was removed from the list of resistance-inducing mutations.^31^

The remaining sites discovered by our method yet not included in the expert rules^5^,^6^,^30^ deserve further attention. Indeed, mutations at sites 208, 218 and 228 have even been previously suspected^32^ to contribute to resistance.

The presented predictive models are derived from a large, although limited number of training examples. Even a very large number of examples would not guarantee that they cover all possible sorts of mutations. A particular advantage of rough sets is the ability to deal with contradictions. A rule that classifies an object to e.g. the “susceptible OR resistant” class is actually very useful since it indicates that, with the present knowledge, the object can belong any of these classes. If such rule has a significant coverage, it suggests the directions of further research. This ability is especially important in the context of medical applications where it is more desirable to perform additional examination than misclassifying the case.

While statistically sound, our findings should be subjected to further experimental validation and we see them as a navigational aid for clinicians and molecular biologists.

## Conclusion

The presented approach led us to the *in silico* discovery of several previously unknown mutations that contribute to resistance to RT inhibitors. Moreover, we discovered the exact values of the biochemical properties that will lead to resistance. This extends applicability of our model to previously unseen cases. Last, but not least, this approach can be applied to a wide class of similar problems, such as analysis of influenza neuramidase-mutants resistant to drugs, protein engineering or efficient drug design.

## Figures and Tables

**Table 1.** Number of resistance-annotated sequence examples per class.

**Class**	**Drug**	**Number of examples**								**Total**
		**Training set**				**Test set**				
		**Susceptible**	**Moderately resistant**	**Resistant**	**Total**	**Susceptible**	**Moderately resistant**	**Resistant**	**Total**	
NRTI	Abacavir	159	257	150	566	39	64	37	140	706
	Didanosine	271	256	40	567	67	63	9	139	706
	Lamivudine	172	95	307	574	42	23	76	141	715
	Stavudine	295	182	89	566	73	45	22	140	706
	Tenofovir	183	61	31	276	46	15	7	68	344
	Zidovudine	274	143	147	564	68	35	36	139	703
NNRTI	Delavirdine	352	95	132	579	87	23	33	143	722
	Nevirapine	316	43	240	599	79	10	59	148	747

**Table 2.** Physicochemical descriptors of amino acids used in this study.

**No.**	**aaIndex^11^ code**	**Abbreviation**	**Descriptor**
1	RADA880102	E oct-wat.	Transfer free energy from octanol to water^14^
2	FAUJ880103	vdW vol.	Normalized van der Waals volume^15^
3	ZIMJ680104	isoel. P	Isoelectric point^16^
4	GRAR740102	polarity	Polarity^17^
5	CRAJ730103	freq. turn	Normalized frequency of turn^18^
6	BURA740101	freq. helix	Normalized frequency of alpha-helix^19^
7	CHAM820102	E sol. wat.	Free energy of solution in water^20^

**Table 3.** Sites selected by the MCFS as significant for resistance to Abacavir (NRTI). Only the top-scoring property is presented per site. Prevalence of mutations in the data and MCFS score are reported.

**Rank**	**Site**	**Property**	**Score**	**Prevalence**	**Status**	
1	P184	E sol. wat.	104.39	0.57	Known for NRTIs (abacavir, didanosine, lamivudine)	*
8	P210	freq. helix	66.11	0.26	Known for NRTIs (abacavir, stavudine, tenofovir, zidovudine)	*
12	P41	isoel. point	41.61	0.4	Known for NRTIs (abacavir, didanosine, stavudine, tenofovir, zidovudine)	*
16	P215	E oct-wat.	34.39	0.54	Known for NRTIs (abacavir, didanosine, stavudine, tenofovir, zidovudine)	*
27	P67	vdW vol.	18.34	0.11	Known for NRTIs (abacavir, stavudine, tenofovir, zidovudine)	*
32	P151	freq. turn	14.55	0.04	Known for NRTIs (abacavir, didanosine, lamivudine, stavudine, zidovudine)	*
33	P75	vdW vol.	14.12	0.09	Known for other NRTIs (stavudine)	+
36	P74	polarity	13	0.11	Known for NRTIs (abacavir, didanosine, tenofovir)	*
37	P219	freq. helix	12.79	0.27	Known for other NRTIs (didanosine, stavudine, zidovudine)	+
39	P118	E oct-wat.	12.48	0.17	Known but considered unimportant	*
41	P44	vdW vol.	12.18	0.1	Known for other NRTIs (tenofovir)	+
49	P43	freq. helix	10.61	0.14	Unknown	+++
54	P116	freq. helix	9.77	0.03	Unknown	+++
59	P115	isoel. point	9.36	0.03	Known for NRTIs (abacavir)	*
78	P228	E oct-wat.	7.99	0.14	Unknown	+++
79	P65	vdW vol.	7.97	0.04	Known for NRTIs (abacavir, didanosine, lamivudine, tenofovir)	*
83	P70	freq. turn	7.6	0.28	Known for NRTIs (didanosine, stavudine, tenofovir, zidovudine)	+
86	P135	freq. turn	7.42	0.42	Unknown	+++
87	P181	freq. helix	7.39	0.15	Known for NNRTIs (efavirenz, etravirine, nevirapine)	++
91	P122	isoel. point	7.28	0.49	Unknown	+++

Symbols represent the status of a site:

* Sites known to contribute to resistance to the particular drug;

+ Sites where mutations are associated with resistance to some NRTI drugs but not to Abacavir;

++ Sites where mutations contribute to resistance to NNRTI drugs;

+++ Sites that are not included in the literature.^5^,^6^,^30^

**Table 4.** Sites selected by the MCFS as significant for resistance to Delavirdine (NNRTI). Only the top-scoring property is presented per site. Prevalence of mutations in the data and MCFS score are reported.

**Rank**	**Site**	**Property**	**Score**	**Prevalence**	**Status**	
1	P151	E oct-wat.	42.48	0.04	Known for NRTIs (abacavir, didanosine, lamivudine, stavudine, zidovudine)	*
4	P184	vdW vol.	36.06	0.56	Known for NRTIs (abacavir, didanosine, lamivudine)	*
7	P41	isoel. point	35.55	0.4	Known for NRTIs (abacavir, didanosine, stavudine, tenofovir, zidovudine)	*
10	P210	E sol. wat.	27.36	0.26	Known for NRTIs (abacavir, stavudine, tenofovir, zidovudine)	+
21	P75	freq. helix	22.54	0.09	Known for NRTIs (stavudine)	+
26	P215	E oct-wat.	20.15	0.54	Known for NRTIs (abacavir, didanosine, stavudine, tenofovir, zidovudine)	*
28	P118	E oct-wat.	18.88	0.17	Known but considered unimportant	*
29	P116	freq. helix	18.38	0.03	Unknown	+++
32	P74	polarity	17.36	0.11	Known for NRTIs (abacavir, didanosine, tenofovir)	*
58	P65	isoel. point	9.51	0.04	Known for NRTIs (abacavir, didanosine, lamivudine, tenofovir)	*
60	P44	E oct-wat.	9.37	0.1	Known for NRTIs (tenofovir)	+
64	P67	vdW vol.	7.94	0.11	Known for NRTIs (abacavir, stavudine, tenofovir, zidovudine)	+
68	P43	freq. helix	7.27	0.14	Unknown	+++
76	P218	polarity	6.61	0.08	Unknown	+++
83	P228	freq. turn	6.23	0.14	Unknown	+++
85	P219	E sol. wat.	5.95	0.27	Known for NRTIs (didanosine, stavudine, zidovudine)	*
86	P211	freq. turn	5.68	0.54	Unknown	+++

Symbols represent the status of a site:

* Sites known to contribute to resistance to Delavirdine;

+ Sites where mutations are associated with resistance to some NNRTI drugs but not to Delavirdine;

++ Sites where mutations contribute to resistance to NRTI drugs;

+++ Sites that are not included in the literature.^5^,^6^,^30^

**Table 5.** Sites selected by the MCFS as significant for resistance to Lamivudine (NRTI). Only the top-scoring property is presented per site. Prevalence of mutations in the data and MCFS score are reported.

**Rank**	**Site**	**Property**	**Score**	**Prevalence**	**Status**	
1	P184	vdW vol.	407.38	0.56	Known for NRTIs (abacavir, didanosine, lamivudine)	*
8	P67	E oct-wat.	25.35	0.11	Known for NRTIs (abacavir, stavudine, tenofovir, zidovudine)	+
12	P41	isoel. point	21.62	0.4	Known for NRTIs (abacavir, didanosine, stavudine, tenofovir, zidovudine)	+
13	P215	vdW vol.	a21.29	0.54	Known for NRTIs (abacavir, didanosine, stavudine, tenofovir, zidovudine)	+
18	P75	freq. turn	18.47	0.09	Known for NRTIs (stavudine)	+
21	P210	E oct-wat.	16.23	0.26	Known for NRTIs (abacavir, stavudine, tenofovir, zidovudine)	+
25	P65	E oct-wat.	14.52	0.04	Known for NRTIs (abacavir, didanosine, lamivudine, tenofovir)	*
44	P44	E oct-wat.	9.4	0.1	Known for NRTIs (tenofovir)	+
50	P118	E oct-wat.	7.07	0.17	Known but considered unimportant	*
51	P228	E oct-wat.	7	0.14	Unknown	+++
57	P83	E sol. wat.	6.03	0.15	Unknown	+++
62	P211	vdW vol.	5.66	0.54	Unknown	+++
64	P70	isoel. point	5.6	0.28	Known for NRTIs (didanosine, stavudine, tenofovir, zidovudine)	+
65	P122	vdW vol.	5.58	0.48	Unknown	+++
66	P181	aisoel. point	5.57	0.15	Known for NNRTIs (efavirenz, etravirine, nevirapine)	++

Symbols represent the status of a site:

* Sites known to contribute to resistance to Lamivudine;

+ Sites where mutations are associated with resistance to some NRTI drugs but not to Lamivudine;

++ Sites where mutations contribute to resistance to NNRTI drugs;

+++ Sites that are not included in the literature.^5^,^6^,^30^

**Table 6.** Sites selected by the MCFS as significant for resistance to Stavudine (NRTI). Only the top-scoring property is presented per site. Prevalence of mutations in the data and MCFS score are reported.

**Rank**	**Site**	**Property**	**Score**	**Prevalence**	**Status**	
1	P215	E oct-wat.	101.72	0.54	Known for NRTIs (abacavir, didanosine, stavudine, tenofovir, zidovudine)	*
3	P210	isoel. point	82.64	0.26	Known for NRTIs (abacavir, stavudine, tenofovir, zidovudine)	*
11	P67	vdW vol.	59.61	0.11	Known for NRTIs (abacavir, stavudine, tenofovir, zidovudine)	*
14	P41	isoel. point	50.64	0.4	Known for NRTIs (abacavir, didanosine, stavudine, tenofovir, zidovudine)	*
27	P151	vdW vol.	26.31	A0.04	Known for NRTIs (abacavir, didanosine, lamivudine, stavudine, zidovudine)	*
29	P75	polarity	22.94	0.09	Known for NRTIs (stavudine)	*
30	P208	isoel. point	22.44	0.1	Unknown	+++
31	P118	freq. helix	22.24	0.17	Known but considered unimportant	*
33	P44	E oct-wat.	21.59	0.1	Known for NRTIs (tenofovir)	+
35	P69	E oct-wat.	21.02	0.15	Known for NRTIs (abacavir, didanosine, lamivudine, stavudine, tenofovir, zidovudine)	*
47	P219	freq. turn	18.15	0.27	Known for NRTIs (didanosine, stavudine, zidovudine)	*
48	P70	vdW vol.	17.83	0.28	Known for NRTIs (didanosine, stavudine, tenofovir, zidovudine)	*
52	P116	polarity	16.93	0.03	Unknown	+++
60	P43	freq. helix	15.78	0.14	Unknown	+++
94	P218	freq. helix	8.31	0.08	Unknown	+++
96	P228	isoel. point	7.83	0.14	Unknown	+++
100	P203	freq. turn	7.45	0.12	Unknown	+++
101	P122	vdW vol.	7.17	0.48	Unknown	+++
103	P184	E sol. wat.	6.63	0.56	Known for NRTIs (abacavir, didanosine, lamivudine)	+
110	P211	polarity	5.9	0.54	Unknown	+++
111	P62	E sol. wat.	5.85	0.05	Part of the multi-nRTi resistance complex. Affects all NRTIs except Tenofovir	*

Symbols represent the status of a site:

* Sites known to contribute to resistance to Stavudine;

+ Sites where mutations are associated with resistance to some NRTI drugs but not to Stavudine;

++ Sites where mutations contribute to resistance to NNRTI drugs;

+++ Sites that are not included in the literature.^5^,^6^,^30^

**Table 7.** Sites selected by the MCFS as significant for resistance to Tenofovir (NRTI). Only the top-scoring property is presented per site. Prevalence of mutations in the data and MCFS score are reported.

**Rank**	**Site**	**Property**	**Score**	**Prevalence**	**Status**	
1	P215	E oct-wat.	37.66	0.53	Known for NRTIs (abacavir, didanosine, stavudine, tenofovir, zidovudine)	*
3	P184	E oct-wat.	26.41	0.49	Known for NRTIs (abacavir, didanosine, lamivudine)	+
10	P67	vdW vol.	17.81	0.12	Known for NRTIs (abacavir, stavudine, tenofovir, zidovudine)	*
13	P210	isoel. point	15.27	0.29	Known for NRTIs (abacavir, stavudine, tenofovir, zidovudine)	*
19	P41	polarity	13.95	0.37	Known for NRTIs (abacavir, didanosine, stavudine, tenofovir, zidovudine)	*
27	P75	E oct-wat.	12.04	0.09	Known for NRTIs (stavudine)	+
30	P203	freq. turn	10.62	0.15	Unknown	+++
31	P65	isoel. point	10.46	0.06	Known for NRTIs (abacavir, didanosine, lamivudine, tenofovir)	*
38	P219	E sol. wat.	9.28	0.31	Known for NRTIs (didanosine, stavudine, zidovudine)	+
47	P43	freq. helix	8.09	0.14	Unknown	+++
48	P44	E oct-wat.	8.07	0.11	Known for NRTIs (tenofovir)	*
56	P35	polarity	6.93	0.28	Unknown	+++
57	P69	vdW vol.	6.89	0.16	Known for NRTIs (abacavir, didanosine, lamivudine, stavudine, tenofovir, zidovudine)	*
58	P101	freq. helix	6.74	0.12	Known for NNRTIs (efavirenz, etravirine, nevirapine)	++
65	P74	E oct-wat.	6.26	0.16	Known for NRTIs (abacavir, didanosine, tenofovir)	*
74	P70	isoel. point	5.85	0.28	Known for NRTIs (didanosine, stavudine, tenofovir, zidovudine)	*
77	P200	polarity	5.38	0.31	Unknown	+++
91	P135	polarity	4.71	0.38	Unknown	+++
94	P208	isoel. point	4.64	0.11	Unknown	+++

Symbols represent the status of a site:

* Sites known to contribute to resistance to Tenofovir;

+ Sites where mutations are associated with resistance to some NRTI drugs but not to Tenofovir;

++ Sites where mutations contribute to resistance to NNRTI drugs;

+++ Sites that are not included in the literature.^5^,^6^,^30^

**Table 8.** Sites selected by the MCFS as significant for resistance to Zidovudine (NRTI). Only the top-scoring property is presented per site. Prevalence of mutations in the data and MCFS score are reported.

**Rank**	**Site**	**Property**	**Score**	**Prevalence**	**Status**	
1	P215	polarity	173.43	0.54	Known for NRTIs (abacavir, didanosine, stavudine, tenofovir, zidovudine)	*
6	P67	isoel. point	78.24	0.11	Known for NRTIs (abacavir, stavudine, tenofovir, zidovudine)	*
11	P41	isoel. point	58.56	0.4	Known for NRTIs (abacavir, didanosine, stavudine, tenofovir, zidovudine)	*
19	P210	isoel. point	41.71	0.26	Known for NRTIs (abacavir, stavudine, tenofovir, zidovudine)	*
25	P70	isoel. point	28.08	0.28	Known for NRTIs (didanosine, stavudine, tenofovir, zidovudine)	*
28	P219	isoel. point	23.91	0.27	Known for NRTIs (didanosine, stavudine, zidovudine)	*
37	P75	polarity	18.15	0.09	Known for NRTIs (stavudine)	+
46	P184	E oct-wat.	13.69	0.56	Known for NRTIs (abacavir, didanosine, lamivudine)	+
48	P69	E oct-wat.	11.88	0.15	Known for NRTIs (abacavir, didanosine, lamivudine, stavudine, tenofovir, zidovudine)	*
56	P151	polarity	9.56	0.04	Known for NRTIs (abacavir, didanosine, lamivudine, stavudine, zidovudine)	*
57	P228	vdW vol.	9.55	0.14	Unknown	+++
62	P43	freq. helix	8.64	0.14	Unknown	+++
63	P203	freq. turn	8.63	0.12	Unknown	+++
64	P116	vdW vol.	8.2	0.03	Unknown	+++
71	P74	isoel. point	7.29	0.11	Known for NRTIs (abacavir, didanosine, tenofovir)	+
72	P44	vdW vol.	7.27	0.1	Known for NRTIs (tenofovir)	+
74	P208	isoel. point	7.21	0.1	Unknown	+++
76	P35	freq. turn	7.05	0.28	Unknown	+++

Symbols represent the status of a site:

* Sites known to contribute to resistance to Zidovudine;

+ Sites where mutations are associated with resistance to some NRTI drugs but not to Zidovudine;

++ Sites where mutations contribute to resistance to NNRTI drugs;

+++ Sites that are not included in the literature.^5^,^6^,^30^

**Table 9.** Sites selected by the MCFS as significant for resistance to Didanosine (NRTI). Only the top-scoring property is presented per site. Prevalence of mutations in the data and MCFS score are reported.

**Rank**	**Site**	**Property**	**Score**	**Prevalence**	**Status**	
1	P103	vdW vol.	134.05	0.07	Known for NNRTIs (efavirenz, nevirapine)	+
8	P181	freq. turn	50.79	0.15	Known for NNRTIs (efavirenz, etravirine, nevirapine)	+
15	P100	E sol. wat.	12.7	0.04	Known for NNRTIs (efavirenz, etravirine, nevirapine)	+
21	P211	isoel. point	7.45	0.51	Unknown	+++
22	P101	vdW vol.	6.71	0.09	Known for NNRTIs (efavirenz, etravirine, nevirapine)	+
23	P190	Polarity	6.62	0.11	Known for NNRTIs (efavirenz, etravirine, nevirapine)	+
26	P74	Polarity	5.61	0.11	Known for NRTIs (abacavir, didanosine, tenofovir)	++
27	P122	Polarity	5.27	0.44	Unknown	++
28	P219	E oct-wat.	5.26	0.25	Known for NRTIs (didanosine, stavudine, zidovudine)	++
29	P210	freq. turn	5.09	0.25	Known for NRTIs (abacavir, stavudine, tenofovir, zidovudine)	++
35	P41	vdW vol.	4.85	0.37	Known for NRTIs (abacavir, didanosine, stavudine, tenofovir, zidovudine)	++
41	P135	E oct-wat.	4.5	0.39	Unknown	+++
49	P184	freq. helix	4.32	0.53	Known for NRTIs (abacavir, didanosine, lamivudine)	++
59	P179	polarity	4	0.13	Known for NNRTIs (etravirine)	+
63	P43	E oct-wat.	3.97	0.13	Unknown	+++
64	P221	polarity	3.97	0.04	Unknown	+++
68	P188	freq. turn	3.77	0.03	Known for NNRTIs (efavirenz, nevirapine)	+
70	P245	freq. turn	3.73	0.32	Unknown	+++
76	P123	E oct-wat.	3.63	0.22	Unknown	+++
87	P67	freq. turn	3.51	0.11	Known for NRTIs (abacavir, stavudine, tenofovir, zidovudine)	++
90	P207	polarity	3.45	0.24	Unknown	+++
96	P200	freq. helix	3.35	0.29	Unknown	+++
100	P35	Polarity	3.33	0.26	Unknown	+++
105	P228	vdW vol.	3.3	0.13	Unknown	+++

Symbols represent the status of a site:

* Sites known to contribute to resistance to Didanosine;

+ Sites where mutations are associated with resistance to some NRTI drugs but not to Didanosine;

++ Sites where mutations contribute to resistance to NNRTI drugs;

+++ Sites that are not included in the literature.^5^,^6^,^30^

**Table 10.** Sites selected by the MCFS as significant for resistance to Nevirapine (NNRTI). Only the top-scoring property is presented per site. Prevalence of mutations in the data and MCFS score are reported.

**Rank**	**Site**	**Property**	**Score**	**Prevalence**	**Status**	
1	P103	vdW vol.	77.84	0.08	Known for NNRTIs (efavirenz, nevirapine)	*
4	P181	freq. turn	57.5	0.15	Known for NNRTIs (efavirenz, etravirine, nevirapine)	*
9	P190	freq. turn	43.42	0.11	Known for NNRTIs (efavirenz, etravirine, nevirapine)	*
22	P100	E sol. wat.	9.99	0.04	Known for NNRTIs (efavirenz, etravirine, nevirapine)	*
23	P101	freq. helix	9.33	0.09	Known for NNRTIs (efavirenz, etravirine, nevirapine)	*
29	P188	vdW vol.	7.95	0.03	Known for NNRTIs (efavirenz, nevirapine)	*
34	P211	isoel. point	6.43	0.52	Unknown	+++
35	P379	E oct-wat.	6.33	0.02	Unknown	+++
36	P98	freq. helix	6.21	0.13	Known for NNRTIs (etravirine, nevirapine)	*
38	P102	E oct-wat.	6	0.15	Unknown	+++
39	P184	E oct-wat.	5.97	0.53	Known for NRTIs (abacavir, didanosine, lamivudine)	++
44	P179	freq. turn	5.66	0.14	Known for NNRTIs (etravirine)	+
46	P74	polarity	5.51	0.11	Known for NRTIs (abacavir, didanosine, tenofovir)	++
51	P106	freq. turn	5.39	0.04	Known for NNRTIs (efavirenz, etravirine, nevirapine)	*
56	P468	E oct-wat.	5.28	0.03	Unknown	+++
63	P357	polarity	4.9	0.06	Unknown	+++

Symbols represent the status of a site:

* sites known to contribute to resistance to Nevirapine;

+ sites where mutations are associated with resistance to some NNRTI drugs but not to Nevirapine;

++ sites where mutations contribute to resistance to NRTI drugs;

+++ sites that are not included in the literature.^5^,^6^,^30^

**Table 11.** Sites mentioned in^5^,^6^ but not selected as significant by the MCFS method are marked with “X”.

**Site**	**Drug**							**Description**
	**ABC**	**ddI**	**3TC**	**d4T**	**TDF**	**AZT**	**NVP**	
P62	X	X	X		X	X		Part of the 69 multi-resistance complex and of the 151 multi-resistance complex. Included in^6^ only.
P69	X	X	X					Part of the 69 multi-resistance complex.
P70		X						Part of the 69 multi-resistance complex and the TAM complex.
P77	X	X	X	X		X		Part of the 151 multi-resistance complex.
P108							X	Included in^6^ only.
P116			X					Part of the 151 multi-resistance complex.
P151			X					Part of the 151 multi-resistance complex.
P219			X					Part of the 69 multi-resistance complex and the TAM complex.
P230							X	Included in^5^ only.

**Abbreviations:** ABC, abacavir; ddI, didanosine; 3TC, lamivudine; d4T, stavudine; AZT, zidovudine; NVP, nevirapine. Delavirdine is not included in the articles.

**Table 12.** Results of the 10-fold cross-validation and the external test obtained by using the set of standard and the set of generalized rules. The underlined value indicates the use of a negated classifier. SD stands for standard deviation and RMSE for root mean squared error (WEKA provides RMSE instead of SD). The highest accuracy and AUC values are in bold.

**Drug**	**Model resistance class**	**CV Accuracy**						**Accuracy (external test set)**			**CV AUC**					**Rules**	
		**Standard**		**General**		**J48**		**Standard**	**General**	**J48**	**Standard**		**General**		**J48**	**Standard**	**General**
		**Mean**	**SD**	**Mean**	**SD**	**Mean**	**RMSE**				**Mean**	**SD**	**Mean**	**SD**	**Mean**	**Number**	**Number**
	**NRTI drugs**																
Abacavir	Susceptible										**0.92**	0.05	0.89	0.07	0.89		
	Intermediate	0.71	0.04	0.7	0.05	**0.72**	0.39	0.61	0.58	**0.65**	**0.76**	0.06	0.75	0.07	0.74	18136	611
	Resistant										**0.84**	0.05	0.82	0.06	0.79		
Didanosine	Susceptible										**0.83**	0.06	0.82	0.06	0.8		
	Intermediate	**0.75**	0.07	0.74	0.06	0.72	0.38	**0.78**	0.73	0.77	**0.8**	0.09	0.79	0.1	0.75	19873	444
	Resistant										**0.91**	0.11	0.9	0.11	0.85		
Lamivudine	Susceptible										**0.95**	0.02	0.94	0.03	0.94		
	Intermediate	0.89	0.03	0.88	0.04	**0.9**	0.24	0.91	**0.93**	0.9	**0.86**	0.1	0.83	0.11	0.83	23994	312
	Resistant										**0.98**	0.02	0.97	0.02	0.97		
Stavudine	Susceptible										0.86	0.07	**0.87**	0.06	0.86		
	Intermediate	0.72	0.04	**0.74**	0.05	**0.74**	0.37	**0.81**	0.8	0.71	0.78	0.06	**0.79**	0.05	0.75	20031	541
	Resistant										**0.93**	0.04	0.89	0.05	0.85		
Tenofovir	Susceptible										**0.87**	0.05	0.86	0.07	0.65		
	Intermediate	**0.78**	0.06	0.76	0.07	0.69	0.41	**0.73**	0.56	0.72	**0.78**	0.05	0.75	0.07	0.6	10078	256
	Resistant										**0.88**	0.09	0.82	0.19	0.74		
Zidovudine	Susceptible										**0.95**	0.03	0.94	0.04	0.89		
	Intermediate	**0.75**	0.04	**0.75**	0.05	0.66	0.42	**0.77**	0.71	0.71	**0.78**	0.06	0.76	0.07	0.63	24975	531
	Resistant										**0.89**	0.06	**0.89**	0.05	0.75		
**NNRTI drugs**																	
Delavirdine	Susceptible										0.78	0.05	0.78	0.04	**0.79**		
	Intermediate	**0.69**	0.06	**0.69**	0.06	**0.69**	0.41	**0.71**	0.67	0.69	**0.61**	0.07	0.6	0.1	0.58	27143	716
	Resistant										**0.81**	0.08	0.8	0.08	0.71		
Nevirapine	Susceptible										**0.87**	0.04	**0.87**	0.04	0.86		
	Intermediate	0.77	0.03	0.78	0.02	**0.83**	0.31	0.76	0.76	**0.77**	0.6	0.16	0.48	0.14	**0.61**	13970	240
	Resistant										**0.87**	0.05	0.85	0.05	0.86		

**Table 13.** The coverage and the accuracy of the rules. For expert rules we compute accuracy and coverage using all the available examples. The “moderately resistant” cases are treated as “resistant”. In the case of rule-based model we compute accuracy and coverage using only the test set examples. This gives pessimistic assessment of both the measures but enables one to avoid possible bias coming from the fact that the rules were derived from the training set. The underlined value indicate that the classifier was negated.

**Drug**	**Expert rules^5^**		**Rough set rule-based model**	
	**Coverage**	**Accuracy**	**Coverage**	**Accuracy**
Abacavir	0.29	0.95	0.85	0.58
Delavirdine	No rules	No rules	0.99	0.67
Didanosine	0.32	0.78	0.99	0.73
Lamivudine	0.58	0.98	1	0.67
Nevirapine	0.4	0.99	0.99	0.76
Stavudine	0.59	0.78	0.98	0.8
Tenofovir	0.44	0.57	0.73	0.56
Zidovudine	0.58	0.83	0.98	0.71
